# Influenza virus infection augments susceptibility to respiratory *Yersinia pestis* exposure and impacts the efficacy of antiplague antibiotic treatments

**DOI:** 10.1038/s41598-020-75840-w

**Published:** 2020-11-05

**Authors:** Yaron Vagima, David Gur, Noam Erez, Hagit Achdout, Moshe Aftalion, Yinon Levy, Ayelet Zauberman, Avital Tidhar, Hila Gutman, Shlomi Lazar, Tomer Israely, Nir Paran, Sharon Melamed, Tal Brosh-Nissimov, Theodor Chitlaru, Irit Sagi, Emanuelle Mamroud

**Affiliations:** 1 Department of Biochemistry and Molecular Genetics, Israel Institute for Biological Research, Ness-Ziona, Israel; 2 Department of Infectious Diseases, Israel Institute for Biological Research, Ness-Ziona, Israel; 3 Department of Pharmacology, Israel Institute for Biological Research, Ness-Ziona, Israel; 4 Infectious Diseases Unit, Assuta Ashdod University Hospital, Ashdod, Israel; 5 Department of Biological Regulation, The Weizmann Institute of Science, Rehovot, Israel

**Keywords:** Antimicrobials, Bacteria, Bacteriology, Clinical microbiology, Infectious-disease diagnostics, Pathogens, Virology, Immunology, Microbiology, Pathogenesis

## Abstract

Various respiratory viral infections in general and seasonal influenza in particular may increase the susceptibility to bacterial infections. Plague caused by *Yersinia pestis* endangers large populations during outbreaks or bioterrorism attacks. Recommended antibiotic countermeasures include well-established protocols based on animal studies and corroborated by effective treatment of human cases. Until now, prior exposure to viral respiratory infections was not taken into consideration when selecting the appropriate treatment for plague. Here, we show that as late as 25 days after exposure to influenza virus, convalescent mice still exhibited an increased susceptibility to sublethal doses of *Y. pestis*, presented with aberrant cytokine expression, and impaired neutrophil infiltration in the lungs. Increased levels of M2 alveolar macrophages and type II epithelial cells, as well as induction in metalloproteases expression and collagen and laminin degradation, suggested that the previous viral infection was under resolution, correlating with enhanced susceptibility to plague. Surprisingly, postexposure prophylaxis treatment with the recommended drugs revealed that ciprofloxacin was superior to doxycycline in mice recovering from influenza infection. These results suggest that after an influenza infection, the consequences, such as impaired immunity and lung tissue remodeling and damage, should be considered when treating subsequent *Y. pestis* exposure.

## Introduction

*Yersinia pestis* is the etiological agent of plague, which has caused millions of deaths throughout human history. The disease has not yet been eradicated, and possible epidemics still constitute an important public health concern, mainly in developing countries. Recently, the island of Madagascar has experienced a severe outbreak of pneumonic plague, making it one of the world’s worst plague epidemics in the last few decades^1,2^. The pneumonic form of plague develops following inhalation of bacteria-containing droplets or aerosols. After an initial flu-like phase, patients develop acute pneumonia and a fulminant disease with the capability of person-to-person transmission^3–5^. High mortality rates in untreated patients as well as the contagious nature of this disease led to the recognition of *Y. pestis* as one of the most dangerous biothreat agents^6^. Several antibiotics are recommended by the Centers for Disease Control and Prevention (CDC) for prophylaxis and treatment of plague, including both bacteriostatic (e.g. doxycycline) and bactericidal drugs (e.g. ciprofloxacin) ^7^.

Influenza viruses are a prevalent cause of seasonal illness associated with upper and lower respiratory tract infections and have been reported as a leading cause of morbidity and mortality, mainly among pregnant women, very young children, the elderly, patients with comorbidities and immunosuppressed individuals^8–10^. It has long been known that influenza increases the susceptibility to secondary bacterial infections caused by *S. pneumonia*, *S. aureus*, *H. influenza* and others^9,11,12^. Recently, an association between severe influenza of immunocompetent patients and invasive Aspergillus infection of the lungs has also been reported, emphasizing the risk of secondary opportunistic infections with influenza^13^ Reports indicate that bacterial coinfections are found in 4–30% of adults and in 22–33% of children who are hospitalized with community-acquired viral pneumonia^14^, and up to 75% of those infected with influenza and develop pneumonia are confirmed to have a bacterial coinfection^11^. Bacterial secondary pneumonia as a complication of viral flu caused by influenza or other respiratory viral pathogens, was reported to occur at very high frequency during seasonal influenza as well as the pandemic of 1918 and multiple subsequent epidemics, for review see^15,16^. Furthermore, a very recent retrospective cohort study of COVID-19 patients from the recent SARS-CoV-2 outbreak in Wuhan, China, indicated that 50 percent of the fatalities may have developed lethal secondary bacterial infections^17^.

Studies using mouse infection models that appear to mimic the clinical course of secondary bacterial infection following influenza indicate that susceptibility to the bacterial infection peaks early after influenza infection^18–20^. The molecular interactions by which influenza viruses are associate with secondary infections involve lung tissue damage and were suggested to be associated with superior access to nutrients and exposure of receptors that enable improved adherence, survival and dissemination to the lower-respiratory tract where resident or invading bacteria are localized^21,22^. Host antiviral responses that might also facilitate secondary bacterial infection also include dysfunction of the early antibacterial innate immune response, leading to suppression of phagocytic and cytotoxic activities by macrophages and neutrophils^23,24^.

Accordingly, in the current study, we reasoned that pre-exposure to seasonal viral respiratory infections may not only increase susceptibility to opportunistic pathogens but also to pneumonia-causing virulent pathogens during a natural outbreak or deliberate events. Furthermore, the present study explores the possibility that suppression of innate immune responses by pulmonary viral infection might continue for long periods of time and reduce the efficacy of antibacterial therapy. Thus, using well-established mouse models, we directly evaluated the influence of influenza on the susceptibility to develop a subsequent plague infection after exposure during a late-stage postinfluenza infection (25 days). The data support the notion that the absence of an adequate inflammatory response concomitant with substandard neutrophil infiltration and bacterial clearance in the lungs was associated with a resolution and tissue remodeling processes; this phenomenon was depicted by an elevation in the number of alveolar macrophages (AMs) harboring the M2 phenotype, the induction of matrix metalloprotease (MMP) expression and extracellular matrix (ECM) remodeling and the elevation in type 2 epithelial cells near the damaged alveolus. This ongoing process and the consequent inferior immune response have an important impact on treatment with the CDC-recommended antibiotic doxycycline (Doxy), but not ciprofloxacin (Cipro), against pulmonary *Y. pestis* infection during late-stage postinfluenza.

## Results

### Enhanced susceptibility to *Y. pestis* infection postinfluenza is associated with a decreased inflammatory response in the lung

**Figure 1:**
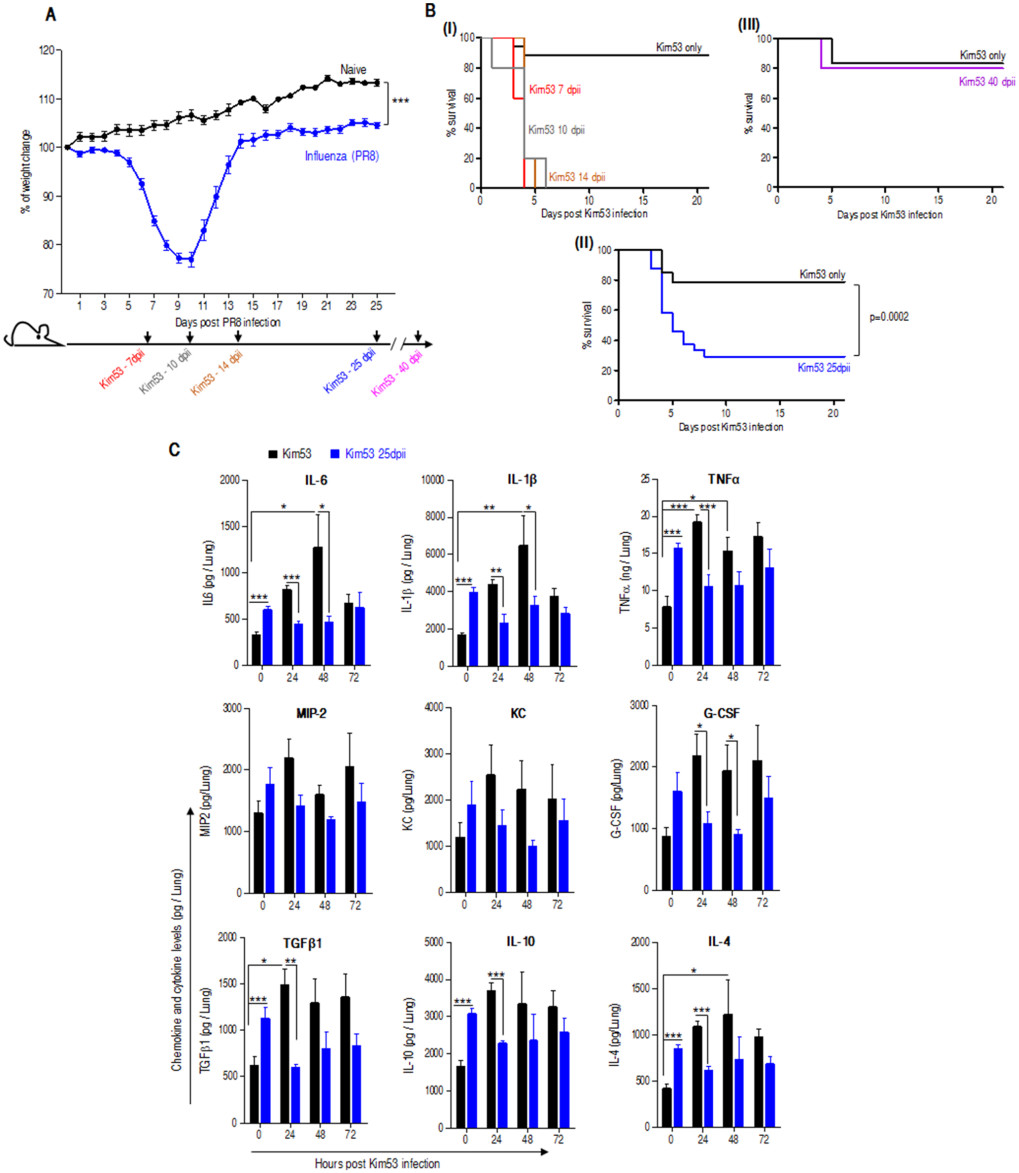
Mouse survival and cytokine expression in the lungs of influenza convalescent mice after airway exposure to Kim53. (**A**) Changes in body weight of mice inoculated i.n. with the PR8 mouse-adapted influenza virus (20 pfu) compare to naïve (n = 24 for mice 25 dpii and n = 9 for naïve mice) ***p < 0.001 vs. naïve. Lower panel, experimental setting scheme of Kim53 infection at different time points postinfluenza. (**B**) Kaplan–Meier survival curves of mice i.n. infected with a sublethal dose (0.2 LD_50_) of the fully virulent *Y. pestis* strain Kim53 at different time points postinfluenza (n = 38 for naïve mice, n = 24 for mice 25 dpii, n = 5 for mice infected with Kim53 at 7, 10, 14 and 40 dpii). (**C**) Cytokine and chemokine levels in the lungs at the indicated time points after i.n. infection of naïve mice with 0.2 LD_50_ of Kim53 (black) compared to mice infected with a similar dose of Kim53 administered at 25 dpii (blue) (n = 7–8 mice per time point,). #*p* < 0.05, ###*p* < 0.001 vs. Kim53 at t = 0. *p < 0.05, **p < 0.01, ***p < 0.001 vs Kim53 at the same time point.

In this study, the well-established murine model of influenza infection based on the mouse-adapted human virus strain A/Puerto Rico/8/1934 (H1N1) influenza virus—PR/8 was employed^19,25^. As depicted in Fig. 1A, mice infected with a sublethal dose of influenza virus exhibited a severe reduction in body weight (~ 25%) 8–10 days postinfluenza infection (dpii), yet all mice survived the infection. The weight loss was followed by a gradual return to the normal weight 13–14 dpii with a stable and moderate elevation in the body weight thereafter, as compare to naïve mice that presented continuous increase in their body weight. To address the question of whether influenza infection results in enhanced susceptibility to pulmonary infection with the *Y. pestis* strain Kim53, mice were infected by i.n. exposure with 0.2 LD_50_ *Y. pestis* at various time points follow influenza infection. *Y. pestis* infection with this low dose was resulted with survival of 80% of naïve mice, which were not preinfected with influenza virus (Fig. 1B-II). As expected, mice showed high susceptibility to a sublethal challenge of *Y. pestis* during the early (7 dpii), mid (10 dpii) and late stages of the disease up to 14 dpii a time point where their initial body weight was regained (Fig. 1B-I). Surprisingly, augmented susceptibility was observed even at 25 dpii, when the virus was effectively cleared from mouse lungs (Figure S1). Forty days postinfluenza infection, mice susceptibility to *Y. pestis* exposure returned to the level observed in naïve mice (Fig. 1B-III). The unexpected susceptibility observed 25 days postinfluenza recovery was the subject of this study, and accordingly, all experiments documented in the present report focus on this time point. We first evaluated cytokine expression in the lungs due to their role in host defense as leukocyte chemoattractants and as activating factors involved in modulation of the innate response to invading pathogens^26^. As depicted in Fig. 1C, naïve mice that were challenged with a sublethal dose of Kim53, exhibited gradual induction in the level of various proinflammatory cytokines and chemokines after bacterial infection. In contrast, mice challenged at 25 dpii had an initial higher cytokines and chemokines levels as compare to naïve mice, however these levels were not increased after *Y. pestis* infection, correlating with their high susceptibility. These observations were supported by appropriate ANOVA (Figure S2). The overall findings motivated inspection of immune cell recruitment to the lung and the consequent bacterial clearance.

### Limited neutrophil infiltration and *Y. pestis* clearance in the lung postinfluenza infection

**Figure 2:**
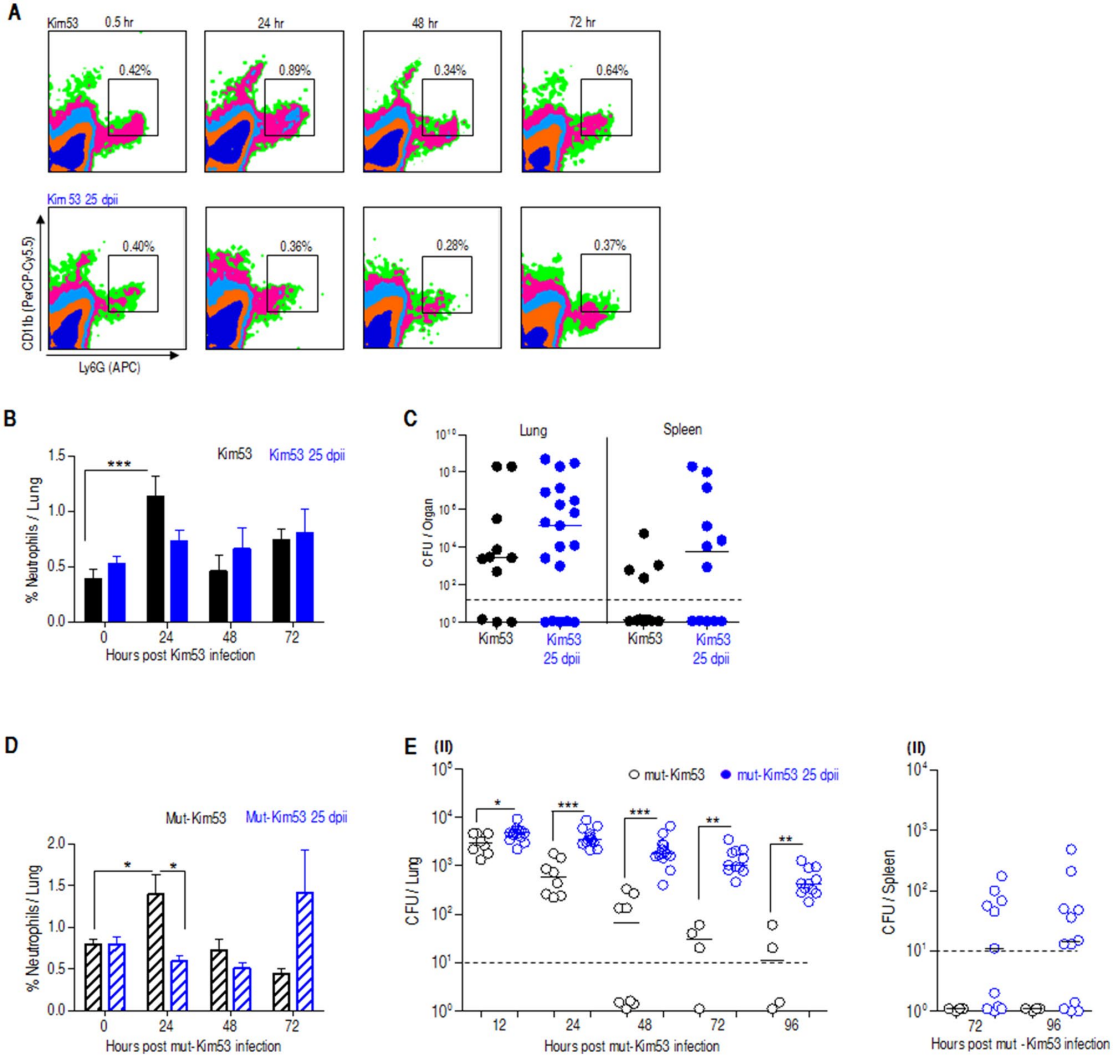
Delayed neutrophil infiltration to the lung and impaired bacterial clearance in mice infected with *Y. pestis* strains 25 dpii. Mice were i.n. infected with a dose of 0.2 LD_50_ of Kim53 (black) or with a similar dose of Kim53 administered 25 dpii (blue). Representative FACS plots (**A**) and a summary of neutrophil percentage in the lungs (**B**) of naïve or 25 dpii mice at the indicated time points after infection with Kim53 (n = 6–8 mice). (**C**) Bacterial loads in the lungs and spleens of naïve or influenza convalescent mice at 72 h after i.n. infection with Kim53 (n = 11–19 mice per group). (**D**) Neutrophil percentage in the lungs of naïve mice or 25 dpii at the indicated time points after infection with a mut-Kim53 (n = 4–11 mice). (**E**) Bacterial loads in the lungs (I) and spleens (II) of naïve mice infected i.n. with a mut-Kim53 strain (black) or with a similar dose administered at 25 dpii (blue). (n = 4–13 mice per group). Dotted lines denote the detection cut-off of 10 cfu per organ. #*p* < 0.05, ###*p* < 0.001 vs. Kim53 at t = 0. *p < 0.05, **p < 0.01, ***p < 0.001 vs mut-Kim53 at the same time point.

Neutrophil recruitment to the site of infection is a fundamental step towards bacterial clearance from infected organs. We have previously shown that the early recruitment of neutrophils to the lung has a beneficial effect on bacterial clearance and mouse survival following *Y. pestis* strain Kim53 infection^27^. Hence, we examined the level of neutrophils in the lungs of both naïve mice and mice infected with influenza 25 days earlier. We detected an induced recruitment of neutrophils to the lungs of naïve mice as early as 24 h after infection with Kim53, while examination of Kim53-infected mice at 25 dpii revealed no elevation in neutrophil level in the lungs (Fig. 2A,B). The lack of infiltrating neutrophils was correlated with a higher bacterial load in the lungs and subsequent dissemination to the spleen (Fig. 2C). To confirm that susceptibility to a sublethal dose of *Y. pestis* postinfluenza infection resulted from an aberrant immune response in the lungs, mice were infected with the avirulent *Y. pestis* strain Kim53∆70∆10 in which the pPCP1 and pCD1 virulence plasmids were cured (mut-Kim53)^27^. While naïve mice were able, as expected, to clear bacteria rapidly from the lungs preventing bacterial dissemination to the blood and spleen, mice previously infected with influenza virus exhibited delayed clearance of the mutated-Kim53 strain from the lungs and spleen (Fig. 2E). As observed with the wild-type *Y. pestis* strain Kim53, clearance of the mutated bacteria from the lungs of naïve mice was associated with neutrophil infiltration to the lung 24 h postinfection, while in influenza-infected mice, the response was delayed and detected only 72 h postinfection (Fig. 2D). Analysis of this data was supported by appropriate ANOVA (Figure S2). Taken together, the data strongly support the notion that the enhanced susceptibility to pulmonary *Y. pestis* exposure, 25 days after influenza infection, involves an attenuation of the innate host immune response in the lungs. It is interesting to note that unlike the innate immune response, the adaptive humoral immune response was not affected, as demonstrated by the observation that the challenge with the mut-Kim53 strain resulted in elicitation of IgG titers targeting *Y. pestis* capsular protein—F1, similar in the sera of naïve mice and influenza-infected mice (Figure S3).

### Enhanced neutrophil recruitment to the lung improves *Y. pestis* clearance but not mouse survival after influenza infection

**Figure 3:**
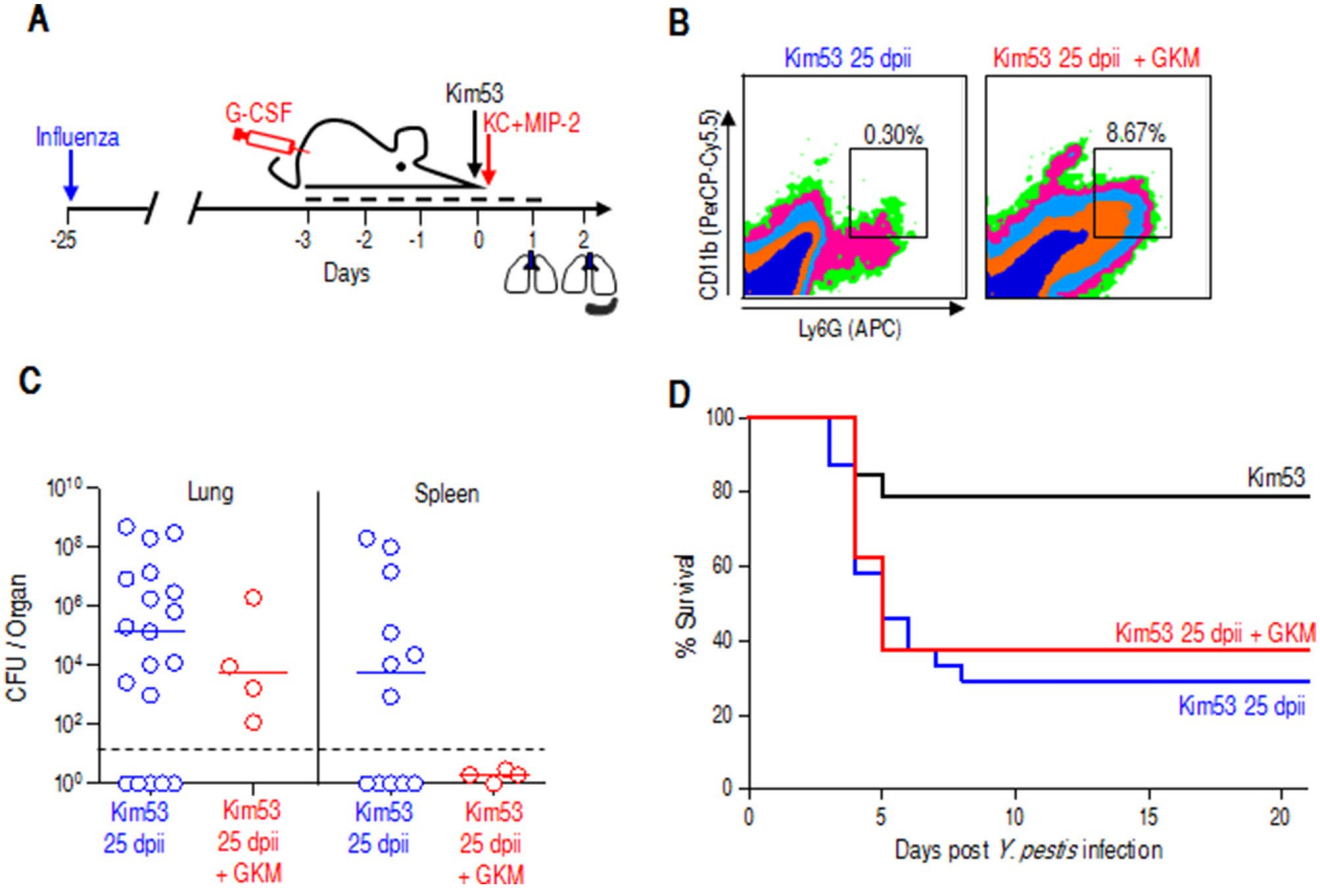
Early recruitment of neutrophils to the lung improves bacterial clearance but not survival rates. (**A**) Experimental setting scheme of combined treatment with G-CSF, KC and MIP-2 (GKM) against Kim53 infection at 25 dpii. (**B**) Representative FACS plots of neutrophils in the lungs of GKM-treated compared to untreated, 24 h after *Y. pestis* infection and 25 dpii. (**C**) Bacterial counts in the lungs and spleens of GKM-treated mice (red circles) compared to in those of untreated mice (blue circles) at 48 h postinfection with *Y. pestis*. n = 11–19 mice per group from 3 independent experiments for Kim53 infection, and 4 mice per group from a single experiment for GKM treatment. Dotted lines denote the detection cutoff of 10 cfu per organ. The inner line indicates the median. Notably, bacterial counts in the lungs and spleens of Kim53 at 25 dpii mice (blue) were adopted from Fig. 2C. (**D**) Kaplan–Meier survival curves of GKM-treated mice (red line) or untreated mice (blue line) after Kim53 infection at 25 dpii. n = 8 for mice infected with Kim53 at 25 dpii + GKM. Survival curves of naïve mice infected with Kim53 (black) or with Kim53 at 25 dpii (blue) were adopted from Fig. 1B.

We previously observed a robust egress of neutrophils from the bone marrow early after pulmonary *Y. pestis* strain Kim53 infection but limited infiltration of these cells into the lung, which could be attributed to insufficient chemoattractant expression in the lung^27,28^. Therefore, we employed the previously documented exogenous chemoattractant treatment to promote the recruitment of circulating neutrophils to the lungs of Kim53-infected mice postinfluenza infection. Accordingly, following influenza infection, mice were treated with a daily subcutaneous injection of G-CSF for 5 consecutive days, starting 3 days prior to a challenge with a sublethal dose of Kim53. Six hours after bacterial infection, recombinant KC and MIP-2 proteins were administered i.n. (Fig. 3A). Indeed, the treatment substantially increased neutrophil levels in the lungs of mice challenged with Kim53 25 dpii (Fig. 3B). This was accompanied by a reduction in bacterial counts in the lungs and their absence in the spleens (Fig. 3C). However, unexpectedly, no improvement was observed in the survival rate of treated mice (Fig. 3D). Taken together, the data indicate that the susceptibility of mice promoted by influenza infection cannot be solely attributed to the observed inadequate neutrophil recruitment and possibly involves other lung resident cells whose activities may have been affected and failed to be restored to their normal level even 4 weeks postinfluenza infection.

### Increased populations of AMs harboring the M2 phenotype and type II epithelial cells indicate an ongoing resolution process postinfluenza infection

**Figure 4:**
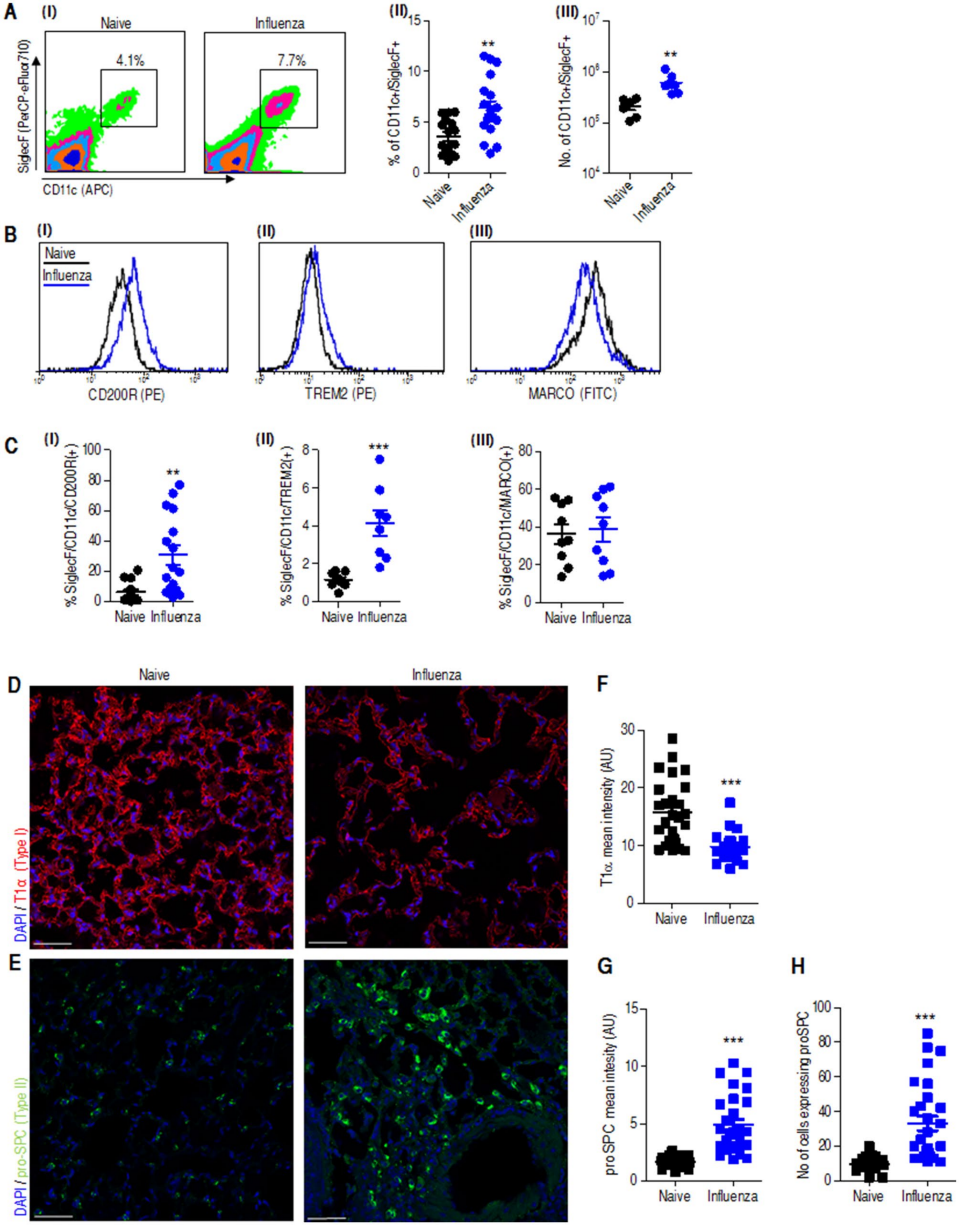
A continuing lung resolution process at 25 dpii characterized by AMs and type II epithelial cell phenotype in the lung. Mice were infected i.n. with a dose of 20 pfu of the influenza virus PR8 (data labeled as blue) or administered with PBS (data labeled as black) and analyzed after 25 days. (**A**) (I) Representative FACS plots of SiglecF/CD11c-positive AMs extracted from whole lungs of influenza-infected mice (25 dpii) compared to those extracted from whole lungs of naïve mice, followed by a summary of alveolar macrophage percentages (II) and absolute numbers (III). n = 16–19 (II) and 6–7 (III) mice per group. (**B**) Representative flow cytometry histograms and (**C**) percentage of SiglecF/CD11b AMs expressing CD220R (I) TREM-2 (II) MARCO (III) in the lungs of PBS (black) and 25 dpii (blue). n = 8–16 mice per group. Immunofluorescence images of lungs naïve or influenza infected lungs (25 dpii) immune-stained for T1α (**D**) and proSPC (**E**). Mean fluorescence intensity quantification of T1 α (**F**) and proSPC (**G**) and the number of proSPC-positive cells (**H**) per field of view as quantified using Zen1 software (Zeiss). Graphs show the mean ± SEM intensity of 10–14 fields per mouse. Scale bar, 50 µm. **p < 0.01, ***p < 0.001 vs naïve.

AMs play an important role as a first line of defense against pulmonary invading pathogens^29^. Evaluation of the percentage and absolute number of AMs revealed an increase in the lung 25 dpii (Fig. 4A). Macrophages can be divided into two functional groups: M1 macrophages that possess proinflammatory properties and mediate host defense against invading pathogens and M2 macrophages that possess antiinflammatory activity and regulate tissue healing^30–33^. We further quantified these two cell populations by cell sorting analysis based on specific immune staining of their surface markers: macrophage receptor with collagenous structure (MARCO) which is highly expressed on M1-macrophages, and CD200 receptor (CD200R) and triggering receptor expressed on myeloid cells (TREM2) which characterize M2-macrophages as previously documented in various models of pulmonary infections^29,34–36^. Expression levels of these surface proteins on CD11c + /SiglecF + AMs as detected by flow cytometer analysis revealed a reduction in MARCO expression and increased cell surface levels of CD200R and TREM2 in mice infected 25 days earlier with influenza virus as compare to naive (Fig. 4B,C). This pattern of surface marker staining indicates that the analyzed AMs belong to the M2 class.

During lung injury, damaged alveolar type (AT) I cells that cover 95–98% of the alveolar surface lead to increased permeability of the lung, while the cuboidal alveolar type (AT) II cells re-epithelialize the alveolar surface via proliferation and transdifferentiation into alveolar type I (ATI) cells as part of the lung repair mechanism^37,38^. We therefore analyzed ATI and alveolar type II (ATII) cell distribution in the lungs at 25 dpii by immune staining of the ATI cell marker T1α and the prosurfactant protein C (proSPC), a marker for ATII cells^37^. Indeed, damaged ATI cells were observed in the lungs as indicated by faint and truncated staining of T1α lining the alveolar septa (Fig. 4D,F). Conversely, the levels of ATII cells were elevated, as indicated by robust proSPC expression 25 dpii (Fig. 4E,G,H). Data analysis was supported by appropriate ANOVA (Figure S2). In conclusion, the increased population of M2 AMs and the high proportion of ATII cells at this late-stage postinfluenza infection strongly suggest that an ongoing tissue repair process is still active in the lungs of the recovered mice and may be responsible for the observed increased susceptibility to a secondary bacterial infection.

### ECM remodeling and MMP expression in the lungs are induced 25 dpii

**Figure 5:**
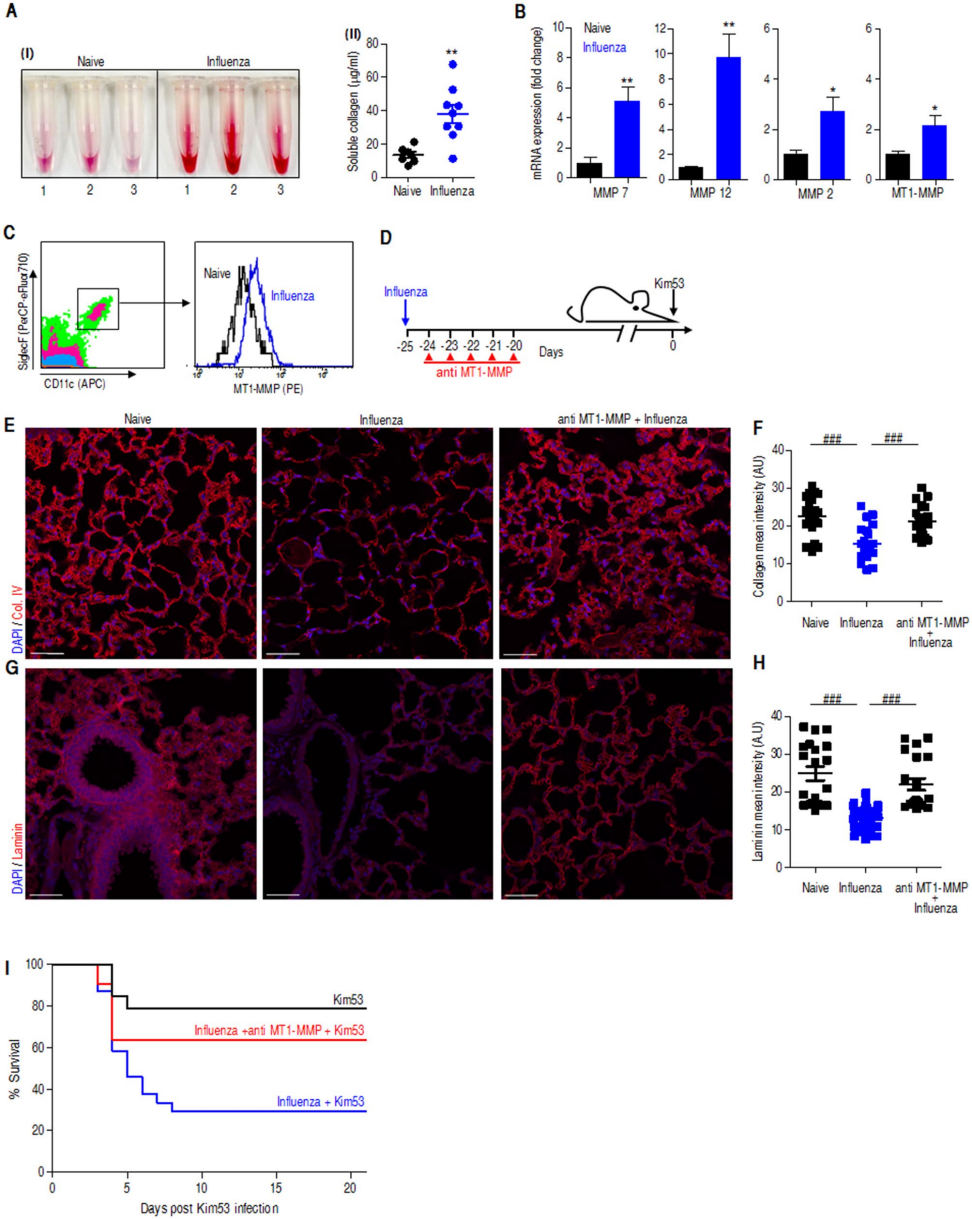
Increased ECM remodeling and MMP expression in the lungs 25 dpii. (**A**) (I) Soluble collagen accumulation in the BALF samples of mice 25 days after i.n. influenza infection. (II) Quantification of collagen concentrations in the BALF samples; n = 7 (naïve) and 9 (influenza). (**B**) qRT-PCR analysis of MMP mRNA in the lungs of mice 25 dpii (blue) compare to naïve mice (black). The data presented as mean ± SEM (n = 7–8). (**C**) Representative FACS analysis of MT1-MMP expression on SiglecF/CD11c AMs extracted from whole lungs of influenza-infected mice (25 dpii) (blue) compared to naïve (black). (**D**) Chronological scheme of treatment with anti MT1-MMP antibody prior to *Y. pestis* exposure. Immunofluorescence images of lungs of naïve or influenza virus infected lungs (25 dpii) and anti MT1-MMP treated mice postinfluenza infection (anti MT1-MMP + Influenza), immunostained for collagen type IV (Col. IV) (**E**) and laminin (**G**). Mean fluorescence intensity quantification of collagen type IV (**F**) and laminin (**H**) as quantified using Zen1 software (Zeiss). Graphs show the mean ± SEM intensity of 10–14 fields per mouse. Scale bar, 50 µm. (**I**) Kaplan–Maier survival curves of anti-MT1-MMP-treated mice (red), untreated mice (blue) and naïve mice (black) challenged i.n. with 0.2 LD_50_ of Kim53 administered at 25 dpii. The survival curves of naïve (black) and influenza convalescent mice (blue) are adapted from Fig. 1B. *p < 0.05, **p < 0.01 vs naïve. ###*p* < 0.001 between naïve vs. influenza and influenza vs. anti-MT1-MMP + influenza.

Tissue repair is a multistep process that includes inflammation, re-epithelialization, and angiogenesis, which are orchestrated by extra cellular matrix (ECM) remodeling. As a prerequisite step during tissue remodeling and repair, ECM proteolysis within the wounded environment involves the expression and activation of various matrix metalloproteases (MMPs) with diverse targets for proteolytic activity, including ECM proteins, adhesion molecules, growth factors, chemokines and cytokines, involving a range of repair processes^39–41^. To further address the tissue repair process in the mice 25 days following influenza infection, we first determined the level of the major ECM component, collagen, in its soluble form in the BALF samples as an indication of ECM proteolysis. As depicted in Fig. 5A, collagen degradation was considerably elevated in the influenza convalescent mice. To substantiate these results, expression levels of various MMPs that play a major role during lung injury were analyzed in the lungs and included MMP7, MMP12, MMP2 and MT1-MMP^42^. The data depicted in Fig. 5B indicate that the MMP expression levels were higher in 25 dpii mice than in naïve mice. MT1-MMP was shown to be essential in the ECM remodeling process during influenza or tuberculosis infections^20,43^. In line with this notion, we observed increased MT1-MMP expression in immune cells and AMs in particular during the late stages of postinfluenza infection (Fig. 5C). Accordingly, we questioned whether MT1-MMP blocking would have any therapeutic value in our model. Hence, we applied a therapeutic regime demonstrated to be effective in downregulating MT1-MMP-mediated proteolysis, consisting of treating the mice for five consecutive days starting 24 h after influenza infection, with the selective allosteric and high specific inhibitory antibody (anti-LEM2/15) targeting MT1-MMP proteolytic activity (Fig. 5D). Targeting MT1-MMP activity resulted in a decreased level of tissue proteolysis as well as decreased levels of collagen (Fig. 5E,F) and laminin (Fig. 5G,H) degradation. In support of these observations, after a challenge with a sublethal dose of *Y. pestis,* compared to their untreated counterparts, treated mice with anti MT1-MMP Ab exhibited improved survival (Fig. 5I). These results strongly support the notion that the active resolution process in the lungs of mice is responsible for the susceptibility of the mice to subsequent plague infection. These observations were supported by appropriate ANOVA (Figure S2).

### Postexposure prophylaxis (PEP) antibiotic treatment with ciprofloxacin but not doxycycline is effective against plague after an influenza infection

**Figure 6:**
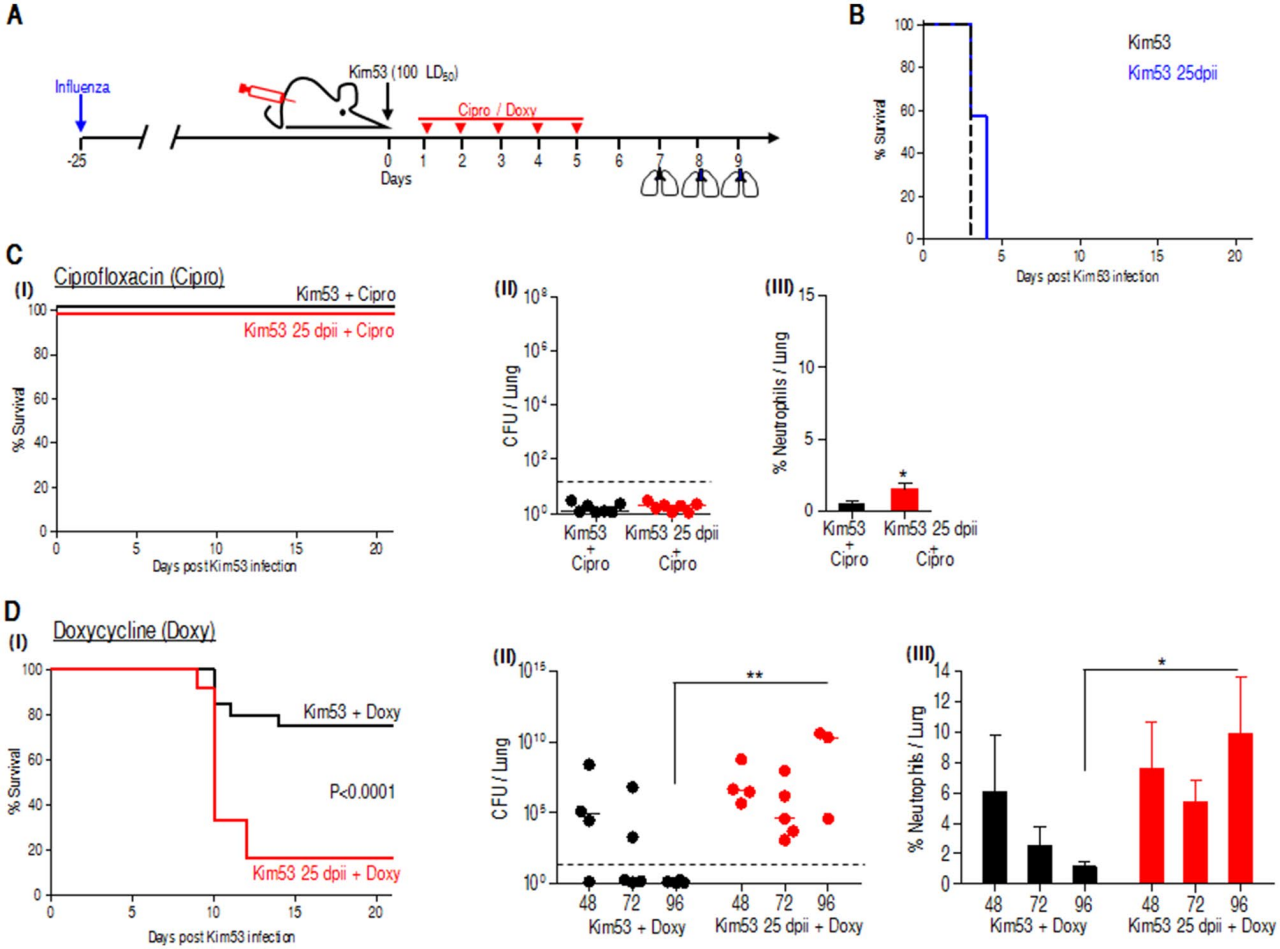
Antibiotic treatment with ciprofloxacin compared to doxycycline against pulmonary *Y. pestis* infection, 25 days postinfluenza infection. (**A**) Experimental scheme for antibiotic treatment: naïve mice were i.n. infected with a lethal dose of 100 LD_50_ Kim53 or with a similar dose of Kim53 administered 25 dpii. Antibiotic treatment was started 24 h after *Y. pestis* exposure for 5 consecutive days with Cipro (40 mg/kg q 24 h) or with Doxy (50 mg/kg q 12 h). (**B**) Kaplan–Maier survival curves of mice i.n. infected with Kim53 (black) or with Kim53 at 25 dpii (blue). Kaplan–Maier survival curves of mice infected i.n. with Kim53 (black) or with Kim53 at 25 dpii (blue) and treated with Cipro (**C-I**) or Doxy (**D-I**) n = 8 and n = 12–20 from 2 and 3 independent experiments for Cipro and Doxy, respectively. Bacterial loads in the lungs of infected mice treated with Cipro (**C-II**) or Doxy (**D-II**) at the indicated time points after the last antibiotic treatment. Dotted lines denote the detection cutoff of 10 cfu per organ. The inner line indicates the median. Neutrophil percentage in the lungs of infected mice treated with Cipro (**C-III**) or Doxy (**D-III**) at the indicated time points after the last antibiotic treatment. n = 7–12 from 2 (for Cipro) or 3 (for Doxy) independent experiments. The data presented as mean ± SEM. **p < 0.01 vs Kim53 + Cipro or Kim53 + Doxy at the same time point.

Both doxycycline and ciprofloxacin are recommended for PEP to treat plague^44,45^. Ciprofloxacin, a fluoroquinolone, is a bactericide that affects bacteria by interfering with DNA replication^46^. Doxycycline, on the other hand, is a bacteriostatic antibiotic that belongs to the tetracycline family, attenuating bacterial growth by targeting the ribosomal subunit 30S, leading to impeded bacterial protein synthesis^46^. Both drugs are effective against *Y. pestis* infection, yet their efficacy has not been evaluated on the background of pulmonary influenza infection. To test their protective potential against *Y. pestis* infection postinfluenza, mice were i.n. infected with a high lethal dose of 100 LD_50_ of the *Y. pestis* strain Kim53 25 dpii. Antibiotic treatment was initiated 24 h after *Y. pestis* infection for 5 consecutive days as described (see Fig. 6A—experimental scheme). In this pneumonic mouse model, nontreated animals succumbed to the infection within 3–4 days (Fig. 6B). Interestingly, while both antibiotics exhibited high protection levels in Kim53 infected mice, doxycycline treatment of *Y. pestis* infected mice postinfluenza infection, exhibited inferior protection capability (20% survival) compared to the full protection achieved by ciprofloxacin treatment (100% survival) (Fig. 6D-I and C-I, respectively). Analysis of bacterial propagation revealed the efficacy of ciprofloxacin in bacterial clearance from the lungs (Fig. 6C-II). On the other hand, doxycycine, which was effective in naïve mice (as reflected by the gradual reduction in bacterial counts), failed to improve bacterial clearance from the lung postinfluenza, and an increase in bacterial counts was observed after the last treatment (Fig. 6D-II). In line with these observations, neutrophils in the lungs of ciprofloxacin-treated mice were slightly higher between naïve and postinfluenza-infected *Y. pestis* mice (Fig. 6C-III). In contrast, doxycycline treatment of naïve mice infected with a lethal dose of Kim53 was followed by neutrophil recruitment to the lungs and their subsequent gradual reduction in correlation to bacterial count. However, neutrophils recruitment that was observed after doxycycline treatment postinfluenza infection was again not efficient in bacterial clearance (Fig. 6D-III), emphasizing the recovery phase of the lungs and the lack of assistance from other immune resident cells.

## Discussion

The causative agent of plague, *Y. pestis*, remains clinically important due to the worldwide distribution of natural plague foci, as well as due to its potential malicious use as a bioweapon^45^. Respiratory tract viral infection has been recognized in recent times as an important predisposition factor for subsequent bacterial pneumonia, which represents a challenging clinical complication^12,23^. Occurrence of natural or bioterrorism-related plague after influenza should not be regarded as a hypothetical situation, as regular seasonal influenza attack rates were shown to be 15.2–22.5% among children and 3.5–10.7% among adults in data reported for unvaccinated controls in controlled vaccine studies during regular seasons^47,48^. Attack rate during influenza pandemics, such as the 2009 H1N1 influenza A are significantly higher, with almost 50% of children infected^49^. Therefore, any outbreak of plague during or after the influenza season would necessarily include individuals recovering from influenza. In this study, for the first time, we investigated a phenomenon of lethal synergism observed in influenza-infected convalescent mice subsequently exposed to *Y. pestis* strain Kim53 pulmonary infection. The study was prompted by the observation that mice exhibited high susceptibility to pulmonary infection with a sublethal dose of *Y. pestis* strain Kim53 almost a month after an influenza infection and, most importantly, almost 2 weeks following an apparent full recovery from disease as observed by return to their initial body weight (Fig. 1A). While various bacterial superinfection on the background of viral influenza is a matter of intense research, most of the studies focused on the early stages of the disease, e.g., 3–7 days postinfluenza viral infection^19,20,50^. Disease manifestation post influenza infection in mice, reaches its peak 7–10 days postinfection as reflected by loss of body mass (Fig. 1A). At this culminant phase of the disease, expectedly, impaired immune responses and severe tissue damage may have explained hyper-susceptibility to a secondary bacterial infection. Here, we documented that, even 25 dpii, the mice remained susceptible to pulmonary infection with a sublethal dose of Kim53. It is important to note that at this stage, mice regained and even exceed their initial body weight and did not express any clinical symptom such as hunched posture, eyes closed, soiled hair coat, nor viral traces could be detected in their lungs; therefore, they appeared to have fully recovered. The study provides evidence that at this late-stage postinfection: (i) the innate immune response in the lungs of mice is still affected; (ii) the lungs are undergoing an alveolar healing process, which correlates with the increased susceptibility to secondary Kim53 infection; and (iii) antibiotic treatment with the bacteriostatic drug doxycycline was found to be considerably less effective for the treatment of plague infection in influenza convalescent mice compared to in naïve animals, a phenomenon that was not observed with the bactericidal drug ciprofloxacin.

### The lung innate immune response elicited in influenza convalescent mice by a secondary *Y. pestis* infection was not optimal 25 days following the primary infection

This conclusion was initially suggested by the profile of inflammatory cytokine expression in the lungs after *Y. pestis* strain Kim53 infection (Fig. 1C). Cytokines constitute the largest and most pleiotropic group of mediators that initiate pulmonary innate responses including mononuclear cell recruitment to inflamed organs. While their immediate increase in the lungs of naïve mice was early observed after infection with a sublethal dose of *Y. pestis*, an absence in elevation were observed in subjects with a previous influenza infection. Notably, relatively higher levels of chemokines and cytokines which are remnants of the inflammatory storm related to viral infection, were detected in convalescent mice at 25 dpii. Immediately after infection, neutrophils are required for reduction in the bacterial load. It is well established that neutrophils have a pivotal role in the response to pulmonary *Y. pestis* infection^27,51–53^. At different stages during pneumonic plague, variable manifestations of neutrophil activity were described. It was shown that neutrophil recruitment to the lung is inhibited during the first 24–36 h after pulmonary infection with a lethal dose (100 LD_50_) of *Y. pestis*^27,54,55^. In this study, pulmonary infection of naïve mice with a sublethal dose of *Y. pestis* strain Kim53 (0.2 LD_50_) was followed by a moderate and transient increased levels of infiltrating neutrophils in the lung, correlating with improved bacterial clearance. Neutrophil data was collected as a proportion; yet, overall neutrophil levels increase was similar to our previous studies and we observed an overall increase in WBC. However, after infection with the sublethal dose of *Y. pestis* carried out 25 days postinfluenza, an increased bacterial propagation in the lung and the spleen was detected, in line with a lower neutrophil recruitment to the lung. A previous study documented a sustained desensitization to bacterial Toll-like receptor ligands after resolution of respiratory influenza infection, an effect that lasted for several months and could be associated with reduced chemokine production and a subsequent limited neutrophil recruitment to the lung^56^. Given the possibility that the lack of neutrophil infiltration to the lungs may be the result of such an inferior Toll-like receptor sensitivity, we implemented an artificial method for rapid induction of neutrophil recruitment to the lung based on combined G-CSF, KC and MIP-2 treatment^27^. Indeed, the treatment resulted in an increase in neutrophil levels in the lung as well as reduced bacterial loads (Fig. 3), yet no improvement in the mouse survival rate was observed. Therefore, we concluded that the observed increased susceptibility of the mice to *Y. pestis* strain Kim53 infection so long after influenza infection cannot be solely attributed to suboptimal neutrophil recruitment. Furthermore, inspection of secondary *Y. pestis* infection with a nonvirulent mutant strain (Fig. 2D,E) indicated that the observed increased susceptibility of convalescent mice did not involve an enhancement of the intrinsic virulence of the bacteria but rather an inferior innate immune response in the lungs.

### Lungs of influenza convalescent mice undergo an extended phase of alveolar restoration, which correlates with increased susceptibility to secondary *Y. pestis* infection

The first line of defense in the lung relies on an immediate sentinel response promoted by perpetual self-renewing resident AMs^57^. In line with their key role, depletion of AMs 3–7 dpii and their subsequent renewal 7 days later were reported^58^. Interestingly, a substantial increase in the population of AMs was observed in the current study 25 dpii (Fig. 4A). Previous studies indicated a reduction in proinflammatory M1 macrophages and a parallel increase in antiinflammatory M2 macrophages during resolution of airway inflammation postinfluenza^29^. In line with the role of M2 macrophages in lung tissue healing, in the current study, we detected the phenotypic CD200R/ TREM2/ MARCO signature of M2 macrophages^34–36^ in the lungs of the convalescent mice (Fig. 4B–C). These data strongly suggest that a prolonged repair mechanism is active 25 days postinfection, much later than anticipated.

Influenza-associated lung injury damages the epithelial endothelial barrier in the alveolus, and this damage is repaired in the course of a healing process of lung resolution dictated by ATII cells expressing surfactant protein C. These self-renewing cells represent precursors of differentiated ATI cells that comprise the majority of the alveolus in the healed lung^59^. The observed reduction in ATI cells, the loss of the epithelium in the bronchioles and the expansion of ATII cells during late-stage postinfluenza infection (Fig. 4D–G) support the notion that a resolution process is in progress in the mouse lungs. Another hallmark of tissue restoration in general and of the lung in particular is the remodeling of collagen and other ECM components by MMPs, which are essential for the repair process^30,42^. MMP proteolytic activities in the lung following damage have intricate and often contradictory roles associated both with deleterious and remodeling effects in the tissue, as well as with modulation of the innate immune response. Their beneficial role in tissue regeneration requires a delicate balance between their levels of expression at various stages of the infection. Interestingly, blocking proteolytic activity in vivo was suggested to have a therapeutic effect in other primary and secondary models of bacterial infections^20^.

During late-stage postinfluenza infection, we determined increased collagen degradation in the BALF samples, accompanied by an MMP expression profile that included upregulation of MMP7, MMP12, MMP2 and MT1-MMP (Fig. 5A–C) pointing on tissue remodeling. The importance of MMP7 during lung re-epithelialization was shown in Mmp7^−/−^ mice subjected to lung injury^60,61^. Similarly, the induction of MMP12 expression in the lungs postinfluenza is in line with its profibrotic protease activity as demonstrated in other lung injury models^62,63^. MT1-MMP is a major collagenase of the lung whose activity is essential for maintaining tissue integrity and is necessary for immune cell recruitment and cytokine expression^42^. Accordingly, in the process of lung healing, its activity is increased, resulting in the depletion of its substrates collagen and laminin (Fig. 5E–H). Furthermore, we show that interfering with MT1-MMP in the course of influenza infection (by antibody-mediated reversal of its activity) improved the survival rate of convalescent mice subjected to secondary pulmonary sublethal *Y. pestis* infection to levels commensurate with those observed in naïve mice (Fig. 5I). These findings are in line with the study that elegantly demonstrated that controling MT1-MMP dysregulated activity during a specific therapeutic window, appears to improve therapeutic effect^20^. The data strongly support the conclusion that the increased susceptibility to plague is associated with physiological lung resolution processes, which are active late in the course of postinfluenza healing. Of note, alveolar cytolysis and acute lung injury are associated with human and rhesus macaques infections by MERS-CoV and SARS^64^, strongly suggesting that a lung healing process, as described in the present report, may represent a frequent cause for secondary bacterial infection following exposure to a wide variety of respiratory viruses including coronavirus.

### Low efficacy of antibiotic treatment with doxycycline for treatment of secondary plague infection

The vulnerability of the murine lungs postinfluenza infection and the observed increase in the susceptibility of the mice to plague prompted us to directly address the possible consequence of this phenomenon on the efficacy of postexposure therapeutic approaches.

Plague can be treated successfully with various antibiotics, including streptomycin, gentamicin, doxycycline and ciprofloxacin, which exploit distinct antimicrobial effects^44,45^. Both doxycycline and ciprofloxacin are recommended as the preferred choice for post-exposure prophylaxis by the CDC^3^. In this study, we found a major difference between the ability of doxycycline and ciprofloxacin to protect mice exposed to pneumonic plague nearly a month after an influenza infection (Fig. 6). The bactericidal activity of ciprofloxacin was efficient in bacterial elimination and led to complete protection against a lethal infection dose of *Y. pestis* strain Kim53 in both naïve and influenza convalescent mice. In contrast, doxycycline treatment resulted in poor survival of similarly infected mice 25 dpii. We speculate that the bacteriostatic nature of this antibiotic heavily depends on the activity of phagocytic cells to manifest its therapeutic potential. Thus, in the context of doxycycline treatment, an optimal operating immune response may be required for full eradication of the metabolically inert, yet live, bacteria, which may not be the case with ciprofloxacin treatment, which directly results in bacterial killing. We cannot rule out additional possible explanations including differential pharmacokinetic properties of the antibiotics, differential immunomodulatory effects or that the healing process taking place in the lungs may have generated an environment that restricts the interaction between the drug and the bacteria. The exact mechanism for the observed limited efficacy of doxycycline after influenza requires additional analysis, and this issue is currently being further studied in our laboratory. Interestingly, such a prolonged period of hyper-susceptibility to secondary bacterial infections was recently documented in COVID-19 hospitalized non-survivor patients, which exhibited secondary infection 17 days after illness onset. While 98% of the patients received antibiotic treatment known to represent an efficient anti-bacterial countermeasure, only 50% survived^17^. In another retrospective study of 85 fatal cases of COVID-19 from Wuhan, combination of anti-microbial drugs did not offer considerable benefit to patients belonging to high risk groups of individuals of advanced age and/or suffering of co-morbidities^65^ These reports, as well as the observations documented in the present report, substantiate the possible effect of viral infection on the efficiency of antibiotic treatment of subsequent bacterial secondary infections, and strongly suggest that such scenarios may require adjustments of the recommended treatments.

Regardless of the mechanisms underlying the phenomena described here, the results underline the importance of the choice of antibiotics against various bacterial infections secondary to seasonal viral influenza as well as other pulmonary viral infections, and shed light on the importance of lungs ECM protection to provide a better outcome of antibiotic treatment. Although plague is a rare bacterial infection, it is clinically relevant in both natural and bioterrorism scenarios, and seasonal influenza infections should be taken into consideration when selecting the appropriate therapeutic response.

## Materials and methods

### *Y. pestis* strains and culture conditions

The fully virulent *Y. pestis* Kimberley53 (Kim53) strain and the avirulent Kimberley53∆70∆10 strain (spontaneously pPCP1- and pCD1-cured Kim53 strain—mut *Y. pestis*) were grown on brain heart infusion agar (BHIA, BD) for 48 h at 28 °C. For intranasal (i.n.) infection, bacterial colonies were harvested and diluted in heart infusion broth (HIB, BD) supplemented with 0.2% (+) xylose and 2.5 mM CaCl_2_ (Sigma-Aldrich) to an OD_660_ of 0.01 and grown for 22 h at 28 °C in a shaker (100 rpm) as previously described^66^. At the end of the incubation period, the cultures were washed, diluted in a saline solution to the required infection dose and quantified by counting colony forming units after plating and incubating on BHIA plates (48 h at 28 °C).

### Ethics statement

All animal experiments were performed in accordance with Israeli law and were approved by the Ethics Committee for animal experiments at the Israel Institute for Biological Research (permit numbers: IACUC-IIBR M-65-2016, and IACUC-IIBR M-90-2016). During the experiments, mice were monitored daily. Humane endpoints were used in our survival studies. Mice exhibiting loss of the righting reflex were euthanized by cervical dislocation. Analgesics were not used as they may have affected the experimental outcomes of the studies.

### Infection of mice with influenza

Strain A/Puerto Rico/8/1934 (H1N1) influenza virus (PR/8) was a kind gift from Dr. Michal Mandelboim (Central Virology Laboratory, Ministry of Health, Chaim Sheba Medical Center, Tel-Hashomer, Israel). The virus was propagated in embryonated chicken eggs as previously described^67^. Briefly, the PR/8 virus was propagated by injecting 0.1 ml of stock virus (hemagglutination unit (HAU = 2,048) diluted 1/100 into the allantoic sac of 11-day-old embryonated chicken eggs. After incubation for 72 h at 37 °C and 30 min at − 20 °C, ~ 8 ml of virus-rich allantoic fluid was removed, HAU was determined with chicken erythrocytes, and virus titers were determined by a plaque assay on Madin–Darby canine kidney (MDCK) cell monolayers. The virus was then stored at − 80 °C until use. For infection, mice were anesthetized with a mixture of 0.5% ketamine HCl and 0.1% xylazine and then infected intranasal (i.n.) with 20 pfu in a volume of 50 μl/mouse.

### Infection of mice with *Y. pestis*

Female C57BL/6 (8–10 weeks old) mice were purchased from Jackson Laboratories (USA) and maintained under defined flora conditions at the Israel Institute for Biological Research animal facilities. i.n. infections were performed as described previously^27^. Briefly, bacterial colonies were harvested and grow as described above. At the end of the incubation period, the cultures were washed, diluted in PBS solution to the required infection dose and quantified by counting colony forming units after plating and incubating on BHIA plates (48 h at 28 °C). Prior to infection, mice were anesthetized with a mixture of 0.5% ketamine HCl and 0.1% xylazine and then i.n. infected with 35 μl/mouse of bacterial suspension, whereas control naïve mice were i.n. instilled with only PBS. The i.n. LD_50_ of the Kim53 strain under these conditions was 1100 cfu. LD_50_ values were calculated according to the method described by Reed and Muench^68^.

### Monitoring bacterial dissemination

To examine bacterial dissemination in the lungs and the spleen, the mice were euthanized, and the lungs and spleens were then removed and placed on a 70 µm nylon cell strainer (BD Falcon) dipped in 2 ml or 1 ml PBS (accordingly) containing a 1% protease inhibitor cocktail (SIGMA). Cell suspension is prepared by mashing the organ against the cell strainer using a syringe plunger^69^. Bacterial quantification was carried out by counting colony forming units after plating and incubating on BHIA plates (48 h at 28 °C).

### Chemokines and cytokine analysis

Chemokines and cytokines analysis was performed as previously described^27^. Briefly, bronchoalveolar lavage fluid (BALF) was collected by exposing the trachea and injecting, then removing, a total of 1 ml PBS containing 1% protease inhibitor cocktail (SIGMA) twice. The BALF samples were then filtered and stored at − 80 °C. Before analysis, samples were centrifuged at 13,000 g for 5 min. The IL-6, IL-1β, TNFα, KC, MIP-2, G-CSF, TGFβ1, IL-10 and IL-4 levels in the BALF were measured by enzyme-linked immunosorbent assay (ELISA) according to the manufacturer’s protocol (R&D Systems).

### Flow cytometry analysis

To generate a lung cell suspension, mice were euthanized, and blood was withdrawn from the mouse hearts with a heparinized syringe. The lungs were then removed and placed on a 70 µm nylon cell strainer as previously described^69^. Cell suspensions were pelleted at 260 g for 10 min at 4 °C. Cells were then fixed in 4% paraformaldehyde in PBS for 1 h at room temperature and washed twice in FACS buffer. Neutrophils (CD11b^+^/Gr-1^high^) were stained with PerCP-Cy5.5-conjugated anti-mouse CD11b (clone M1/70) (eBioscience) and APC-conjugated anti-mouse Ly6G (Gr-1) (clone RB6-8C5) (eBioscience) antibodies. AMs (SiglecF/CD11c) were stained with PerCP-efluor710-conjugated anti-mouse SiglecF (clone 1RNM44N) (eBioscience), APC-conjugated anti-mouse CD11c (clone N418) (eBioscience), PE-conjugated anti-mouse CD220R (clone OX110) (eBioscience), FITC-conjugated anti-mouse MARCO (cat# FAB2956F) (R&D), and PE-conjugated anti-mouse TREM-2 (cat# FAB17291P) (R&D) antibodies. Cells were stained using standard protocols and appropriate matched isotype control antibodies. The analysis was performed on a FACSCalibur flow cytometer with CellQuest Pro (BD Biosciences).

### Combined treatment with G-CSF, KC and MIP-2

Three days prior to infection with *Y. pestis* Kim53, mice received a daily subcutaneous injection of recombinant G-CSF (300 µg/kg rhG-CSF/Neupogen 48 MU/0.5 ml, Roche Applied Science) for 5 consecutive days. Six hours post i.n. infection with *Y. pestis* Kim53, mice were anesthetized, and 1 µg of each recombinant KC and MIP-2 (recombinant MCXCL1/KC and recombinant MCXCL2/MIP-2, R&D Systems**)**, diluted in 25 µl of PBS, or 25 µl of PBS alone (sham) were instilled i.n. Mice were sacrificed and analyzed 24 or 48 hpi or their morbidity and mortality rates were determined.

### Anti MT1-MMP treatment

LEM 2/15 (anti-MT1-MMP) purification was conducted as previously described^20^. Antibody was given for five consecutive days, 24 hpii, 3 mg/kg intraperitoneally, 100 µl per injection.

### RT-PCR and quantitative PCR analysis

Quantitative real-time PCR analysis was performed as previously described^27^. Briefly, total RNA from lung cell suspensions was extracted using TRI Reagent (Cat. T9424, Sigma) according to the manufacturer’s instructions. Two micrograms of total RNA were reverse-transcribed using Moloney murine leukemia virus reverse transcriptase and oligo-dT primers (Promega). Quantitative PCR analysis was performed using an ABI 7500 machine (Applied Biosystems) with SYBR Green PCR Master Mix (Applied Biosystems). The fold change in gene transcript quantity compared with hypoxanthine phosphoribosyl transferase (HPRT) was measured using the comparative (− 2^∆∆Ct^) method. Forty cycles of PCR were performed in duplicate for each primer (Table 1).

**Table 1:** Sequences of primers used in this study.

Mouse gene	Forward 5′–3′	Reverse 5′–3′
**MMP7** NM_010810	GGTGAGGACGCAGGAGTGAA	GCGTGTTCCTCTTTCCATATAACTTC
**MMP12** NM_008605	TGAGGCAGAAACGTGGACTAAA	GGGCTCCATAGAGGGACTGAA
**MMP2** NM_008610	ACCATGCGGAAGCCAAGAT	TTAAGGCCCGAGCAAAAGC
**MMP14** NM_008608	CCCAAAAACCCCGCCTAT	TCTGTGTCCATCCACTGGTAAAA
**HPRT-1** NM_013556	AGTACAGCCCCAAAATGG	TCCTTTTCACCAGCAAGCT
**PR/8 HA**	TGCTAAAACCCGGAGACACA	CGGACCCAAAGCCTCTACTC

### Collagen concentration in the BALF—Sircol assay

The Sircol collagen assay (Biocolor, UK) was performed following the manufacturer’s instructions and as was previously described^70^. Briefly, 1 ml of Sirius red reagent was added to each BALF (50 µl) sample and mixed for 30 min. The collagen-dye complex was precipitated by centrifugation at 10,000 g for 10 min, and the pellet was dissolved in the supplied alkaline reagent. Finally, the absorbance of the samples was measured at 540 nm. The collagen concentration of the influenza-infected samples expressed as µg/ml was normalized to that of the littermate naive samples.

### Immunofluorescence staining—floating sections

Immunofluorescence staining using floating sections technique was performed as previously described^71^. Briefly, the mice were euthanized, and the lungs were rapidly removed, postfixed (4 °C) in 4% neutral-buffered paraformaldehyde (pH = 7.0, 4 °C) for 24 h, then immersed in 4% neutral-buffered paraformaldehyde (pH 7.0, 4 °C) containing 30% sucrose until the lungs sunk (approximately 2 weeks) and frozen at − 80 °C until use. Then, 30-μm-thick cryosections were serially cut and transferred to a 12-well culture dish. Three milliliters of PBS were added gently to each well containing a section to allow the sections to thaw and float in the buffer. Sections were washed in PBS for 4 × 15 min (each wash). For immunostaining, sections were stained with the following primary antibodies: polyclonal rabbit anti-collagen IV (Abcam Cambridge, UK), polyclonal rabbit anti-Laminin (Thermo Fischer Scientific, Cheshire, UK), monoclonal hamster anti-Podoplanin (T1α) (Thermo Fischer Scientific, IL, USA) and polyclonal rabbit anti-Pro-SPC (Mercury, CA, USA). Sections were placed in a permeabilization solution (0.2% Triton X-100 in PBS) for 2 h, transferred to a blocking solution [10% normal goat serum (NGS) in PBS containing 0.05% Triton X-100 (TX)] for 4 h, and incubated for 48 h at 4 °C with the primary antibodies. The anti-collagen IV and anti-podoplanin antibodies were diluted 1:500 in primary antibody cocktail (50% blocking buffer in 0.05% TX in PBS); the anti-laminin and anti-pro-SPC antibodies were diluted 1:1000 in a primary antibody cocktail. All primary antibodies were washed for 4 × 20 min in wash buffer (1% blocking buffer and 0.05% TX in PBS); the anti-collagen IV and anti-laminin antibodies were incubated with an Alexa Fluor-594 goat anti-rabbit (1:250) antibody; the anti-podoplanin antibody was incubated with an Alexa Fluor-594 goat anti-hamster (1:250) antibody; and the anti-pro-SPC antibody was incubated with an Alexa Fluor-488 goat anti-rabbit (1:250) antibody for 48 h at 4 °C and washed for 4 × 20 min in wash buffer (the secondary antibodies goat anti-rabbit-Alexa Fluor 488/594 and goat anti-hamster-Alexa Fluor 594 were purchased from Molecular Probes, OR, USA). Sections were further washed with PBS, 3 × 15 min, and then the nuclei were counterstained with DAPI (0.3 µg/ml for 5 min.); washed for 3 × 15 min in PBS and mounted with Fluoromount-G and analyzed using a Zeiss LSM 710 Confocal Microscope (Zeiss, Oberkochen, Germany). The severity score was based on fluorescence intensity quantification of the markers and was performed using Zen1 software (Zeiss).

### Antibiotics and treatment protocols

Ciprofloxacin (Ciproxin, Bayer) was injected subcutaneously according to a regimen of 40 mg/kg once a day for 5 days. Doxycycline (cat# D9891, SIGMA) was injected intraperitoneal according to a regimen of 50 mg/kg every 12 h for 5 days.

### Statistical analysis

Data are presented as mean ± SEM, and differences between groups were assessed by analysis of variance (ANOVA), followed by an appropriate ad hoc analysis (Tukey, Dunnet, student’s *t*-test) using GraphPad Prism 5. A value of *p* < 0.05 was accepted as statistically significance. Statistics are indicated for each result individually in Figure S2.

## Supplementary information

Supplementary Figure 1.

Supplementary Figure 2.

Supplementary Figure 3.
