# Supplementary material for: Influenza virus infection augments susceptibility to respiratory Yersinia pestis exposure and impacts the efficacy of antiplague antibiotic treatments
Source: Sci Rep. 2020 Nov 5;10:19116. doi: 10.1038/s41598-020-75840-w (PMC7645720; doi:10.1038/s41598-020-75840-w)
Supplement: Supplementary file 1 — Supplementary Figure 1. [file 41598_2020_75840_MOESM1_ESM.docx]

**Influenza virus infection augments susceptibility to respiratory *Yersinia pestis* exposure and impacts the efficacy of antiplague antibiotic treatments**

YARON VAGIMA^1*^, DAVID GUR^1^, NOAM EREZ^2^, HAGIT ACHDOUT^2^, MOSHE AFTALION^1^, YINON LEVY^1^, AYELET ZAUBERMAN^1^, AVITAL TIDHAR^1^, HILA GUTMAN^3^, SHLOMI LAZAR^3^, TOMER ISRAELY^2^, NIR PARAN^2^, SHARON MELAMED^2^, TAL BROSH-NISSIMOV^4^, THEODOR CHITLARU ^1^, IRIT SAGI^5^ and EMANUELLE MAMROUD^1*^

**Supplement 1: Influenza virus mRNA levels in mice lungs at 25 days post infection**

**Figure S1.** Ct values of a qRT-PCR analysis of the viral protein Hemagglutinin (HA) cDNA diluted in total whole lung cDNA obtained from naïve mice to identify the limit of detection of viral cDNA in the lungs. A positive signal was not measured beyond 6 decimal dilutions from the initial concentration (9x10^7^ pfu/ml) providing a limit of detection of 90 pfu/ml. NTC represents Non Template Control which was similar in its Ct values (Ct=35) to dilutions 7 and 8. As depicted above, cDNA extracted from the lungs of mice 25 days post influenza infection was below the level of detection as viral HA RNA was not identified. Results are an average of a duplicate with ±SEM.
