# Supplementary material for: Influenza virus infection augments susceptibility to respiratory Yersinia pestis exposure and impacts the efficacy of antiplague antibiotic treatments
Source: Sci Rep. 2020 Nov 5;10:19116. doi: 10.1038/s41598-020-75840-w (PMC7645720; doi:10.1038/s41598-020-75840-w)
Supplement: Supplementary file 2 — Supplementary Figure 2. [file 41598_2020_75840_MOESM2_ESM.docx]

**Influenza virus infection augments susceptibility to respiratory *Yersinia pestis* exposure and impacts the efficacy of antiplague antibiotic treatments**

YARON VAGIMA^1*^, DAVID GUR^1^, NOAM EREZ^2^, HAGIT ACHDOUT^2^, MOSHE AFTALION^1^, YINON LEVY^1^, AYELET ZAUBERMAN^1^, AVITAL TIDHAR^1^, HILA GUTMAN^3^, SHLOMI LAZAR^3^, TOMER ISRAELY^2^, NIR PARAN^2^, SHARON MELAMED^2^, TAL BROSH-NISSIMOV^4^, THEODOR CHITLARU ^1^, IRIT SAGI^5^ and EMANUELLE MAMROUD^1*^

**Supplement 2: Significant outcome of the statistical analyses**

**Figure 1A:** Two ways ANOVA (group x time): group effect: F(1,806)=896.15 p<0.0001, time effect: F(25,806)=39.25 p<0.0001, interaction: F(25,805)=18.19 p<0.0001.

**Figure 1C:** Two ways ANOVA (group x time) followed by *post hoc* one way ANOVA within groups and Tukey between groups:

IL-6: Group effect: F(1,53)=4.9 p<0.03, interaction: F(3,53)=4.82 p<0.048, 1 way ANOVA F(3,26)=3.998 #p<0.05 of 0 vs 48, Tukey *p<0.05 ***p<0.001 compare to Kim53 of the same time point.

IL-1β: Time effect: F(3,52)=3.52 p<0.02, interaction: F(3,52)=5.84 p<0.001, 1 way ANOVA F(3,26)=5.019 ##p<0.01 of 0 vs 48, Tukey *p<0.05 **p<0.01 ***p<0.001 compare to Kim53 of the same time point.

TNFα: Group effect: F(1,55)=3.52 p<0.04, interaction: F(3,55)=8.28 p<0.0001, 1 way ANOVA F(3,26)=5.019 #p<0.05 ##p<0.01 ###p<0.001 of 0 vs 24, 48 and 72 Tukey ***p<0.001 compare to Kim53 of the same time point.

G-CSF: Group effect: F(1,53)=4.14 p<0.04, interaction: F(3,53)=3.01 p<0.03, Tukey *p<0.001 compare to Kim53 of the same time point.

TGFβ1: Group effect: F(1,49)=6.94 p<0.01, interaction: F(3,49)=5.23 p<0.003, 1 way ANOVA F(3,26)=3.132 #p<0.05 of 0 vs 24, Tukey **p<0.01 ***p<0.001 compare to Kim53 of the same time point.

IL-10: interaction: F(3,51)=3.46 p<0.02, Tukey ***p<0.001 compare to Kim53 of the same time point.

IL-4: interaction: F(3,51)=3.10 p<0.03, 1 way ANOVA F(3,27)=2.918 #p<0.05 of 0 vs 48, Tukey ***p<0.001 compare to Kim53 of the same time point.

**Figure 2B:** Two ways ANOVA (group x time) followed by *post hoc* one way ANOVA within groups and Tukey between groups: Time: F(3,69)=4.58 p<0.005. One way ANOVA Kim53: F(3,30)=6.956 ***p<0.001 of 0 vs 24 hr.

**Figure 2D:** Two ways ANOVA (group x time) followed by post hoc 1 way ANOVA within groups and Tukey between groups: Interaction: F(3,30)=3.40 p<0.004. 1 way ANOVA mut-Kim53 F(3,30)=4.58 *p<0.05 of 0 vs 24. Tukey *p<0.05 compare to mut-Kim53 of the same time point.

**Figure 2E:** Two ways ANOVA (group x time) followed by *post hoc* one way ANOVA within groups and Tukey between groups: Interaction: F(4,86)=2.67 p<0.03, time: F(4,86)=23.05 p<0.0001. Tukey *p<0.05 **p<0.01 *p<0.001 compare to mut-Kim53 of the same time point.

**Figure 4A, C, F, G and H:** Two-tailed Unpaired t tests: A: t(33)=.447, p<.0016, C: **p<0.01 ***p<0.001 compare to naïve.

**Figure 5A:** Two-tailed Unpaired t test with Welch's correction **p<0.01 compare to naïve.

**Figure B:** Two-tailed Unpaired t test *p<0.05 **p<0.01 compare to naïve.

**Figure 5F:** One way ANOVA, F(2,64)=17.85 p<0.0001, post hoc (Bonferroni) between naïve vs. influenza and influenza vs. anti-MT1-MMP + influenza ###p<0.0001.

**Figure 5H:** One way ANOVA, F(2,77)=33.99 p<0.0001, post hoc (Bonferroni) between naïve vs. influenza and influenza vs. anti-MT1-MMP + influenza ###p<0.0001.

**Figure 6C (III):** Two-tailed Unpaired t test *p<0.05 compare to Kim53+Cipro.

**Figure 6D (II):** The data was logarithmically transformed and analyzed by Two ways ANOVA (group x time) followed by post hoc (Bonferroni) between groups. F(1,19)=21.75 p<0.01 compare to Doxy+Kim53 at the same time point.

**Figure 6D (III):** Two ways ANOVA (group x time) support these observations followed by post hoc 1 way ANOVA within groups and Tukey between groups: Group: F(1,19)=4.9 p<0.03. Tukey *p<0.05 compare to Kim53+Doxy at the same time point.
