# Supplementary material for: Influenza virus infection augments susceptibility to respiratory Yersinia pestis exposure and impacts the efficacy of antiplague antibiotic treatments
Source: Sci Rep. 2020 Nov 5;10:19116. doi: 10.1038/s41598-020-75840-w (PMC7645720; doi:10.1038/s41598-020-75840-w)
Supplement: Supplementary file 3 — Supplementary Figure 3. [file 41598_2020_75840_MOESM3_ESM.docx]

**Influenza virus infection augments susceptibility to respiratory *Yersinia pestis* exposure and impacts the efficacy of antiplague antibiotic treatments**

YARON VAGIMA^1*^, DAVID GUR^1^, NOAM EREZ^2^, HAGIT ACHDOUT^2^, MOSHE AFTALION^1^, YINON LEVY^1^, AYELET ZAUBERMAN^1^, AVITAL TIDHAR^1^, HILA GUTMAN^3^, SHLOMI LAZAR^3^, TOMER ISRAELY^2^, NIR PARAN^2^, SHARON MELAMED^2^, TAL BROSH-NISSIMOV^4^, THEODOR CHITLARU ^1^, IRIT SAGI^5^ and EMANUELLE MAMROUD^1*^

**Supplement 3: Anti-*Y. pestis* F1 IgG titers in the blood after infection with mut-Kim53**

**
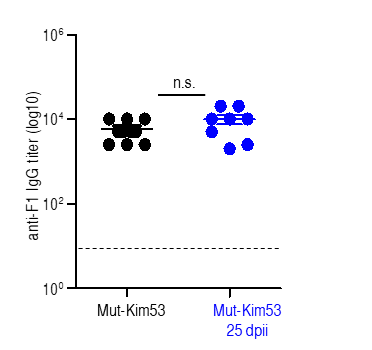
**

**Figure S3. Adaptive immune responses that developed against F1 were similar in naïve mice and mice that recovered from influenza infection.** Anti-F1 IgG titers measured in the sera of naïve mice 30 days after pulmonary infection with mut-Kim53 (n=10; black circles) and in the sera of mice that were similarly challenged with mut-Kim53 25dpii (n=8; blue circles). Dashed line indicates the limit of detection. The inner line represents the mean ±SEM.
